# THE ASSOCIATION OF MORTALITY WITH DEMENTIA IN VETERANS ENROLLED IN A MEMORY DISORDERS CLINIC: THE EFFECT OF FRAILTY

**DOI:** 10.1093/geroni/igz038.3206

**Published:** 2019-11-08

**Authors:** Aakashi Shah, Raquel Apracio-Ugarriza, Amar Morani, Mercedes Rodriguez-Suarez, Jorge G Ruiz

**Affiliations:** 1 Department of Public Health, University of Miami Miller School of Medicine, Miami, Florida, United States; 2 Miami VAHS Mental Health Service, Miami, Florida, United States; 3 Miami VAHS Geriatric Research, Education and Clinical Center, Miami, Florida, United States

## Abstract

Dementia is a syndrome of deterioration in cognition and ability to perform everyday activities. Frailty, a state of vulnerability to stressors leading to increased morbidity, mortality and utilization is a determinant of dementia. The aim was to determine if dementia leads to increased mortality in Veterans and whether frailty affects this association. We conducted a retrospective cohort study of 308 Veterans enrolled in VA memory disorders clinic during 2016-2019. Dementia was diagnosed based on complete clinical assessments, brain imaging and neuropsychological testing. A 44–item frailty index (FI) was constructed using demographics, comorbidities, medications, laboratory tests, and activities of daily living. Patients were divided into non-Frail (FI<0.21) and Frail (FI≥0.21). After adjusting for age, race, ethnicity, income, education, substance abuse, BMI, comorbidities, hospitalizations, medication use, the association of dementia with mortality was assessed using Cox proportional hazards regression. Patients were 55.2% White, 74% non-Hispanic, and the mean age was 74.4 ± 8.3 years. 113 patients were diagnosed with dementia out of which 27 died. Over a median follow-up period of 526 days (Interquartile Range: 431.5 days), there were 27 deaths. There was a significant and positive association of dementia with mortality significant even after all adjustments, HR=2.65 (95% CI: 1.02-6.92), p: 0.045. After subgroup analysis, there was no significant association between mortality and dementia according to frailty status. Our study results suggest that dementia is associated with a higher risk for mortality in Veterans at a Memory Disorders clinic. Frailty did not modify the effect.

